# Living Arrangements and Dementia Among the Oldest Old: A Comparison of Mexicans and Mexican Americans

**DOI:** 10.1093/geroni/igac014

**Published:** 2022-03-17

**Authors:** Phillip A Cantu, Jiwon Kim, Mariana López-Ortega, Sunshine Rote, Silvia Mejia-Arango, Jacqueline L Angel

**Affiliations:** Sealy Center on Aging, University of Texas Medical Branch, Galveston, Texas, USA; Department of Educational Psychology, The University of Texas at Austin, Austin, Texas, USA; National Institute of Geriatrics, National Institutes of Health, Mexico City, Mexico; Department of Population Studies, El Colegio de la Frontera Norte, Tijuana, Baja California, Mexico; Kent School of Social Work, University of Louisville, Louisville, Kentucky, USA; LBJ School of Public Affairs and Center on Aging and Population Sciences, The University of Texas at Austin, Austin, Texas, USA

**Keywords:** Caregiving, International, Latino/a (Mexican American)

## Abstract

**Background and Objectives:**

The growing population of adults surviving past age 85 in the United States and Mexico raises questions about the living arrangements of the oldest old and those living with dementia. This study compares Mexican and Mexican American individuals aged 85 and older to identify associations with cognitive status and living arrangements in Mexico and the United States.

**Research Design and Methods:**

This study includes 419 Mexican Americans in 5 southwestern states (Hispanic Established Population for the Epidemiologic Studies of the Elderly) and 687 Mexicans from a nationally representative sample (Mexican Health and Aging Study). It examines characteristics associated with living alone using logistic regression and describes the living arrangements of older adults with probable dementia in each country.

**Results:**

Older adults with dementia were significantly less likely to live alone than with others in the United States while there were no relationships between dementia and living arrangements in Mexico. However, a substantial proportion of older adults with dementia lived alone in both nations: 22% in the United States and 21% in Mexico. Among Mexican Americans with dementia, those living alone were more likely to be women, childless, reside in assisted living facilities, and less likely to own their homes. Similarly, Mexican individuals with dementia who lived alone were also less likely to be homeowners than those living with others.

**Discussion and Implications:**

Contextual differences in living arrangements and housing between the United States and Mexico pose different challenges for aging populations with a high prevalence of dementia.

**Translational Significance:** The U.S. infrastructure for older adults offers more opportunities for the oldest old to maintain living alone, even in the case of those with serious cognitive decline, and Mexico continues to rely almost exclusively on family care for the oldest old.

Dementia is one of the most common causes of disability and dependence in the world. An estimated 21% of people between ages 85 and 90 and 47% of people over age 90 live with dementia in the United States (Prince et al., 2013). The rapid aging of populations across the globe presents significant challenges related to the care of older adults, especially the oldest old (aged 85+ years). There were an estimated 6.6 million adults over age 85 in the United States in 2019 (Administration for Community Living, 2021). The oldest old are the fastest growing segment of the U.S. population over age 65, and the proportion of Hispanic origin oldest old will increase, from 5.6% in 2012 to an anticipated 12.5% by 2050 (Ortman et al., 2014). The growing dementia population in Mexico is exemplary of many low- and middle-income countries (LMICs; Angel et al., 2017). An estimated 6.1% of older adults over age 60 in Mexico have dementia (Mejia-Arango & Gutiérrez Robledo, 2011). Despite the high levels of potential dependency in these two growing populations, neither have high rates of institutional care (Angel et al., 2022). Instead, both populations tend to continue to live in the community, even with dementia.

While the United States has a highly developed formal long-term care system, the use of institutional and community care among Mexican Americans has been low (Angel et al., 2016; Thomeer et al., 2015). Older Mexican Americans rely heavily on family for assistance even as their physical and mental functioning decline (Angel et al., 1996, 2014; Rote et al., 2019). Indeed, declines in cognitive functioning are predictive of household extension for Mexican Americans (Prickett & Angel, 2017), through both having potential caregivers move in and moving in with potential caregivers (Cantu & Angel, 2017). Hispanics who eventually enter nursing homes with dementia typically do so at more advanced stages of disease progression than non-Hispanic Whites (Rivera-Hernandez et al., 2019). As a result, they live for longer periods in the community with a greater need for assistance.

Older adults who live alone are particularly at high risk for institutionalization. The extent to which Mexican Americans live alone with dementia is unclear, but data from the National Healthy Aging Trends Study of Medicare beneficiaries in the United States indicate that 32% of older adults living with dementia live alone (Amjad et al., 2016). Despite the precarity of living alone with dementia (Portacolone et al., 2019), previous research on the living arrangements of older Mexican Americans has focused on household extension rather than those living alone, and there has been little focus on living alone with dementia.

Mexico lacks a publicly financed long-term care system, and it does not have a national-level mandatory registry of institutions, compulsory standards of care, nor a regulatory body to oversee management, quality of care standards for services, or the accreditation and evaluation of service providers (González-Bautista et al., 2021). There are no policies, public programs, or services to provide dependency care, including support for people living with dementia and their family caregivers. Data from the 2020 Population and Housing Census identified 25,357 older adults living in long-term residential settings (including nursing homes), representing less than 1% of the total population 60 years and older (INEGI, 2021). There are few publicly funded, free-of-charge long-term care options in Mexico. For-profit institutions are expensive and as a result, most older adults are unable to afford them (López-Ortega & Aranco, 2019). These conditions have rendered the family the primary source of care and support for both Mexicans and Mexican Americans in late life. Among all goods and services generated by the health sector in Mexico, unpaid care represents 26.6% of the total gross domestic product, a larger percentage than all hospital services (21.2%) and ambulatory (first-level) services (17.2%; López-Ortega & Aranco, 2019). In 2020, 31% of all Mexican households had at least one adult 60 years and older (INEGI, 2021). At the same time, the number of people living alone in Mexico has increased substantially in the past decade, with adults over 60 making up 41% of people living alone (INEGI, 2021). As in other LMICs, there is limited public support for the aging population in Mexico, leaving families with the main responsibility of providing care and economic security for older adults (Angel et al., 2022; López-Ortega & Aranco, 2019).

Little is known about older adults with dementia who live alone in both populations. Despite the high level of assistance needed for dementia care, research on the living arrangements of community-dwelling oldest old adults with dementia among Mexican Americans and Mexicans is limited. This study examines the association between dementia and living arrangements (alone vs with others) in two different contexts: oldest old adults in Mexico and Mexican-origin oldest old adults in the Southwest United States, using two representative samples: the Hispanic Established Population for the Epidemiologic Studies of the Elderly (H-EPESE; Markides et al., 1999) and the Mexican Health and Aging Study (MHAS; Wong et al., 2017). We focus on dementia as it represents severe cognitive impairment that often requires high levels of care and assistance. This study first describes the demographic profiles of the oldest old in each nation. Second, we examine the characteristics associated with living alone. Finally, we describe the living arrangements of older adults with dementia to profile groups at the greatest risk of institutionalization (in the United States) and death (in both countries).

## Method

### Data

The H-EPESE is a prospective cohort household-based sample that, at baseline, was representative of Mexican Americans aged 65 years and older living in the Southwestern states of Arizona, California, Colorado, New Mexico, and Texas. The original baseline sample of 3,050 was interviewed between September 1993 and June 1994 with follow-up interviews roughly every 2 years, and a secondary refreshing sample of 902 was interviewed during the fifth follow-up between September 2004 and June 2005. The first wave of the H-EPESE utilized a multistage area probability sampling design in the Southwestern states to cover 90% of all Mexican Americans in the United States (see Cantu & Markides, 2019 for a detailed account of how the first wave of the H-EPESE utilized census tracts from 1990 to create a representative sample). Interviews took place both in person and via proxy. For this study, we used the ninth wave (2015/2016) of the H-EPESE with a sample of 480 respondents aged 85 years and older and limited to those with no missing covariates (*n* = 419). The H-EPESE is especially appropriate for looking at dementia and living arrangements among the oldest old, as no other population-representative data set has an adequate sample size to support conclusions about Mexican Americans 85 years and older.

The MHAS is a prospective panel study of adults 50 years and older in Mexico (Wong et al., 2017). The baseline survey was conducted in 2001 to ensure national and urban/rural representation of adults born in 1951 or earlier. Follow-up interviews were conducted in 2003, 2012, 2015, and 2018. In 2012, a new sample of adults was added to ensure representation of adults born between 1952 and 1962. In this study, we used data from the 2015 MHAS. To make the samples comparable, out of the total number of 14,779 respondents 50 years and older in 2015, we restricted our sample to those aged 85 and older (*n* = 733) who had no missing information on covariates (*n* = 687). Detailed descriptions of missingness and sample selection are illustrated in Supplementary Figures 1 and 2.

### Living Arrangements

We created a typology of living arrangements based on the number of people living with the survey respondent and the marital status of the survey respondent: living alone, living with a partner, and extended households. Respondents with no coresidents were classified as living alone (H-EPESE *n* = 119; MHAS *n* = 150). We classified those who lived with their spouse or common-law partner but no one else as living with a partner (H-EPESE *n* = 75; MHAS *n* = 88), and those who lived with someone other than their partner as extended households (H-EPESE *n* = 225; MHAS *n* = 449). Extended households in both nations are comprised of adult children, other family members, and other nonfamily members. Our classification of living arrangements reflects differences in potential sources of care. Older adults who live alone may have fewer potential caregivers, while those living with a partner or in extended households have more potential sources of care (Mudrazija et al., 2020).

### Probable Dementia

Probable dementia is determined using cognitive functioning and disability. In the H-EPESE, we followed previous research (Mejia-Arango et al., 2020) to determine dementia based on standardized scores on the Mini-Mental State Examination (MMSE; Folstein et al., 1975), and four instrumental activities of daily living (IADL): ability to handle money, prepare meals, take medication, and go shopping alone. MMSE standardization is established by level of education (no education, 1–6 years, and 7+ years) and individuals scoring one or more standard deviation below the mean of their education group who also report one or more IADL limitation were considered to have probable dementia. Likewise, older adults who answered the survey with a proxy due to cognitive impairment were also considered to have probable dementia. The MHAS uses the modified Cross-Cultural Cognitive Examination (Glosser et al., 1993) to assess cognitive impairment through direct interviews, which covers 10 cognitive domains. The Informant Questionnaire on Cognitive Decline in the Elderly (IQCODE) is used to assess cognitive status for respondents using a proxy. A cutoff score of 3.4 in the IQCODE is used to classify participants with probable dementia (Cherbuin & Jorm, 2017). Individuals who are impaired in two cognitive domains and with an IADL disability or score of >3.4 in the IQCODE were considered to have probable dementia. IADLs included in the determination of dementia in each population include the ability to prepare meals, handle finances, shop alone for groceries and other necessities, and manage medications (Mejía-Arango et al., 2015). For clarity and conciseness, we refer to older adults with probable dementia as those living with dementia throughout the manuscript.

### Exploratory Variables

We examined the correlates of living alone, including demographic and housing characteristics, poverty, and availability of help. Because the context of living alone varies by nation, the exploratory variables for each nation differ in both conceptualization and operationalization. In both nations, we examined the role of age and gender (ref: male). In the United States, we also controlled for being U.S.-born (ref: born in Mexico). We examined marriage in both nations by comparing the married (including those with domestic partners) to the nonmarried (including the widowed, divorced, and never married). We controlled for education in each nation by using three categories: no formal education, 1–6 years, and 7 years and older, in line with previous research on these populations (Mejía-Arango et al., 2015).

Housing characteristics included homeownership in both nations. Additionally, we controlled for housing types in the United States, categorized as detached single homes, multifamily/apartment homes (which includes condos, apartments, and duplexes/triplexes), and assisted living facilities. In Mexico, we examined detached single homes and multifamily/apartment homes, which included condos, apartments in buildings, and neighborhood apartments. In both surveys, housing type was reported by the interviewer.

In the United States, we examined the role of poverty in living arrangements by controlling for Medicaid program participation. Although some individuals qualify for Medicaid through disability, for most older adults in the United States, qualifying reflects near-poverty levels of wealth and income (Angel et al., 2019). In Mexico, we controlled for poverty by using the bottom 40% of wealth for adults over age 85. While this measure does not necessarily reflect poverty in Mexico, an estimated 41% of adults over age 65 in Mexico live in poverty as defined by official government poverty thresholds (CONEVAL, 2018).

Availability of help was measured by reporting one activity of daily living (ADL) limitation and reporting having someone to help with ADL disability. We also controlled for having any living children in both samples.

### Analytic Plan

We began by describing the characteristics of respondents in each living arrangement category for Mexican Americans and Mexicans separately (Table 1). We then used binomial logistic regression to examine the correlates of living alone compared to living with others (combining living with a partner and extended households), controlling for dementia and other known covariates for each sample separately (Table 2). Finally, we described the characteristics of older persons with dementia by living arrangements for both nations (Table 3). Due to the limited sample size, we combined people living with a partner and extended households into a single group (living with others) for our regression analysis and for comparing living arrangements of older adults with dementia.

**Table 1.** Sample Characteristics by Living Arrangements of Older Adults 85+

Variable	Mexican Americans (H-EPESE)					Mexicans (MHAS)				
	Alone (*n* = 146, 31%)	Living with a partner (*n* = 79, 17%)	Extended households (*n* = 250, 53%)	Total (*N* = 475)	*p* Value	Alone (*n* = 150, 22%)	Living with a partner (*n* = 88, 13%)	Extended households (*n* = 449, 65%)	Total (*N* = 687)	*p* Value
	% or *M*	% or *M*	% or *M*	% or *M*		% or *M*	% or *M*	% or *M*	% or *M*	
Probable dementia (%)	21	21	33	27	.064	39	22	46	41	<.01***
Age (years; *M*)	90.40	89.03	90.68	90.30	<.01***	89.12	88.45	88.96	88.93	.024**
Female (%)	74	39	68	64	<.01***	60	33	59	56	<.01***
U.S.-born (%)	46	53	56	53	.553	—	—	—	—	
Years of education (%)										
0	11	8	19	15	.025**	43	33	43	42	.167
1–6	60	65	52	57	.171	43	43	49	47	.37
7+	29	27	29	29	.515	13	24	8	11	<.01***
Married (%)	10	100	19	31	<.01***	0	100	29	32	<.01***
Bottom 40% wealth (%)	—	—	—	—		50	23	40	40	<.01***
Medicaid (%)	56	43	49	50	.119					
Own home (%)	51	72	60	60	.015	80	94	90	88	<.01***
Type home (%)										
Single-detached home	55	85	91	80	<.01***	—	—	—	—	
Single-family home	—	—	—	—		95	95	98	97	.163
Multifamily/apartment	34	8	9	16	<.01***	5	5	2	3	.163
Assisted living	11	7	0	5	<.01***	—	—	—	—	
ADL help (%)	44	40	51	47	.05**	39	25	39	37	.02**
Has living children (%)	86	99	97	94	<.01***	93	95	95	94	.347

*Notes*: ADL = activities of daily living; H-EPESE = Hispanic Established Population for the Epidemiologic Studies of the Elderly; MHAS = Mexican Health and Aging Study. **p <.01, ***p <.001.

*Sources*: 2015 Hispanic Established Population for the Epidemiologic Studies of the Elderly (H-EPESE) and 2015 Mexican Health and Aging Study (MHAS).

**Table 2.** Logistics Regression Predicting Living Alone (Ref: Living With a Partner or Extended Households) Among Older Adults 85+

Variable	U.S. Mexican Americans (H-EPESE)	Mexico Mexicans (MHAS)
	OR (95% CI)	OR (95% CI)
Dementia	0.53* (0.29–0.97)	0.73 (0.47–1.15)
Age	1.02 (0.96–1.09)	1.01 (0.97–1.06)
Female	1.69* (1.03–2.79)	1.12 (0.76–1.64)
U.S.-born	0.64 (0.38–1.06)	
Years of education		
1–6	1.50 (0.75–3.00)	0.94 (0.63–1.40)
7+	2.04 (0.91–4.59)	1.37 (0.75–2.51)
Medicaid	1.41 (0.87–2.28)	
Bottom 40% wealth		1.50* (1.02–2.20)
Own home	0.70 (0.44–1.13)	0.50** (0.30–0.83)
ADL help	0.91 (0.54–1.53)	1.25 (0.79–1.96)
Has living children	0.17*** (0.07–0.44)	0.81 (0.38–1.71)
Cons	0.24 (0.00–100.63)	0.16 (0.00–12.17)
*N*	475	687
Log likelihood	−231.80	−350.82

*Notes*: ADL = activities of daily living; CI = confidence interval; H-EPESE = Hispanic Established Population for the Epidemiologic Studies of the Elderly; MHAS = Mexican Health and Aging Study; OR = odds ratio. *p <.05, **p <.01, ***p <.001.

**Table 3.** Living Arrangements and Sample Characteristics of Older Adults age 85+ With Probable Dementia

Variable	Mexican Americans (H-EPESE)				Mexicans (MHAS)			
	Alone (*n* = 25, 22%)	Living with others^a^ (*n* = 90, 78%)	Total (*N* = 115)	*p* Value	Alone (*n* = 59, 21%)	Living with others^a^ (*n* = 226, 79%)	Total (*N* = 288)	*p* Value
	% or *M*	% or *M*	% or *M*		% or *M*	% or *M*	% or *M*	
Age (years; *M*)	91.04	91.80	91.63	.308	90.56	89.88	90.02	.339
Female (%)	92	66	71	<.001***	59	63	62	.454
U.S.-born (%)	36	56	51	.097	—	—	—	
Years of education (%)								
0	16	19	18	.938	46	48	48	.654
1–6	48	54	53	.566	42	44	44	.942
7+	36	27	29	.574	12	8	8	.356
Married (%)	16	29	26	.144	0	33	26	<.001***
Medicaid (%)	56	57	57	.74	—	—	—	
Bottom 40% wealth (%)	—	—	—		44	45	45	.981
Own home (%)	32	59	53	.012**	78	89	87	.02**
Type home (%)								
Single-detached home	36	89	77	<.001***	—	—	—	
Single-family home	—	—	—		98	97	98	.666
Multifamily/apartment	32	10	15	<.001***	2	3	2	.666
Assisted living	32	1	8	<.001***	—	—	—	
ADL help (%)	76	87	84	.259	69	66	67	.807
Has living children (%)	80	96	92	.017**	90	93	92	.439

*Notes*: ADL = activities of daily living; H-EPESE = Hispanic Established Population for the Epidemiologic Studies of the Elderly; MHAS = Mexican Health and Aging Study. **p<.01, ***p<.001.

^a^Others includes living with a partner and extended households.

## Results

### Sample Description by Living Arrangements

Table 1 shows the characteristics of living arrangements for Mexican Americans and Mexicans. The description of living arrangements in Table 1 tests for significant differences within nations between types of living arrangements. Differences between nations are descriptive and have not been standardized for age distribution for direct comparisons. More Mexican Americans (28%) lived alone than Mexicans (22%). Overall, more Mexicans had dementia than Mexican Americans (41% vs 27%). The extended household category had the highest proportion of adults with dementia in each sample (33% of Mexican Americans and 46% of Mexicans). A larger portion of women lived alone in both nations. Older adults living alone were the least likely to own homes in both countries; however, homeownership was more common for Mexicans than Mexican Americans. Older adults in Mexico who lived alone were more likely to be in poverty (in the bottom 40% of wealth distribution) than those in other living arrangements. Similarly, for Mexican Americans, those who lived alone were most likely to participate in Medicaid.

In the United States, 80% of the oldest old lived in single-detached homes. Just over half of the people living alone in the United States resided in single-family homes (55%), while 34% resided in apartments or multifamily homes, and 11% resided in assisted living facilities. By comparison, almost all housing (97%) in the Mexican sample was identified as single-detached homes. In terms of support availability, a larger portion of Mexican Americans (47%) reported availability of ADL help than older adults in Mexico (37%). However, Mexican Americans who lived alone reported less availability of ADL assistance than those in extended households. In Mexico, older adults living with a partner reported the lowest availability of ADL assistance. In the United States, older adults who lived alone were the least likely to have living children, although the rate was still high, with 86% of those living alone having children.

### Living Alone for Mexican Americans and Mexicans

Table 2 shows results from logistic regressions modeling living alone (vs living with a partner or extended households). Due to lack of variation, marital status and household type were excluded from the regression analysis for both samples. For older Mexican Americans, those with dementia were less likely to live alone (odds ratio [OR]: 0.53; *p* ≤ .05). Women were significantly more likely to live alone than men (OR: 1.69; *p* ≤ .05). Additionally, having living children was associated with lower odds of living alone (OR: 0.08; *p* ≤ .001).

The Mexican sample showed notable differences from the Mexican American sample. Unlike in the United States, dementia was not associated with living alone in Mexico. Lower wealth was associated with greater odds of living alone (OR: 1.50; *p* ≤ .05), while homeownership was associated with lower odds of living alone (OR: 0.50; *p* ≤ .01). None of correlates of living arrangements were significant in both samples.

### Living Alone With Dementia

We describe the characteristics of older adults with dementia by living arrangements in each nation in Table 3. While the small sample size limits our analysis to bivariate statistics, striking differences emerge between the two samples. For Mexican Americans, women represented 92% of those with dementia living alone compared to 59% of people with dementia living with others. In Mexico, there were no gender differences in living arrangements for older adults with dementia, and almost all older adults living alone were unmarried. A significantly smaller percentage of Mexican Americans living alone with dementia own homes (32%) compared to those living with others (59%). Patterns of homeownership were similar in Mexico, although homeownership was more common. In terms of support availability, nearly a third of Mexican Americans living alone with dementia were in assisted living facilities. Fewer Mexican Americans living alone with dementia had living children compared to those living with others. For the other factors considered (education, wealth, or ADL help), people with dementia living alone did not differ from those living with others in either nation.

## Discussion

This study is the first binational comparison of living arrangements among the oldest old in Mexico and Mexican Americans. Although these two populations share similar cultural attitudes and preferences, our findings highlight significant differences in living arrangements for older adults with dementia. Mexican Americans with dementia were significantly less likely to live alone than with others, but there was no relationship between living arrangements and dementia in Mexico. This difference might be related to the lower proportions of older adults living alone in Mexico in general; 28% and 22% of Mexican Americans and Mexicans, respectively, lived alone. In other words, older Mexican Americans live alone even when very impaired, while fewer older adults in Mexico live alone.

Nonetheless, there are individuals with dementia living alone in both samples. Our sample of the oldest Mexican Americans indicated that 22% of older adults with dementia in the United States live alone, compared to 21% of Mexicans. Although our data do not allow us to determine how older adults with dementia were able to live alone, previous research finds that older adults living alone with dementia are at high risk for unmet needs, including household and self-care tasks (Miranda-Castillo et al., 2010).

Research on adults aged 55 and older living with cognitive impairment in the United States demonstrates that 72% report not receiving help with at least one IADL (Edwards et al., 2020). However, we found that the majority of the people living with dementia over age 85 in both nations received ADL help. This is likely because Mexican Americans with ADL disabilities are less likely to live alone than in extended households (Cantu & Angel, 2017), reflecting a high level of care needed and received in late life. These data reveal high levels of support for individuals with dementia living alone, but further research is needed to gauge the adequacy of support in both nations, including help with specific ADL and IADL activities, which the current studies do not reveal. Additionally, examining caregiving intensity will allow future research to address the adequacy of care for older adults living alone.

Findings presented in this study show that older adults with dementia who live alone may have family members nearby who facilitate their independence. For example, in the Mexican sample, 90% of older adults with dementia living alone had at least one living child. However, results from a Mexican national survey show that, while children declare strong willingness to care for their parents, when asked how much they are able to follow through on taking up caregiving activities, they note that adult children are caring for their parents less than they did in the past (López-Ortega et al., 2015). This decrease is usually due to their increasing responsibilities in the labor force and raising their own family. In the United States, however, fewer older Mexican Americans with dementia who lived alone had living children compared to those who lived with others.

Housing availability and type varied significantly between Mexican Americans and Mexicans. In comparison with the U.S. sample, Mexican older adults had higher rates of homeownership, which reflects different contexts for late-life living arrangements. Homeownership was associated with lower odds of living alone for Mexicans but not significantly related to living alone for Mexican Americans. One explanation for the lack of relationship between homeownership and living alone in the United States is eligibility for Medicaid program participation. An increasing number of states are faced with growing Medicaid expenditures as the number of disabled and older adults in need of long-term care grows (Angel et al., 2019). More and more families will find that they not only may be forced to provide at least some financial support to their parents, but that they will inherit none of their parent’s estate, which must be “spent down” for their parent to receive Medicaid. In essence, eligibility criteria might disincentivize coresidence by making it hard to qualify for benefits, especially for those who own homes. Additionally, growing household wealth in the United States in the 20th century was associated with increases in independent living among widows (McGarry & Schoeni, 2000), patterns which are likely similar for older Mexican Americans. In the United States, wealth “buys” privacy, but because most individuals who live in assisted living facilities do not own their home, neither homeownership nor poverty was significantly related to living alone for Mexican Americans.

We found that one third of older Mexican Americans with dementia who lived alone resided in assisted living facilities. This finding indicates that the availability of different types of housing across countries affects living arrangements. The formal caregiving infrastructure in the United States offers opportunities for cognitively impaired older adults to live independently while older adults in Mexico must rely almost exclusively on living with family, as Mexico lacks a similarly developed long-term care system.

Our examination is one of the first to incorporate assisted living facilities into the study of living arrangements for Mexican Americans and reveals some of the shortcomings of examining living arrangements using household rosters. Previous examination of living arrangements in the H-EPESE has relied exclusively on household roster (Prickett & Angel, 2017) or headship status and household roster (Cantu & Angel, 2017) to create typologies of living arrangements without taking into account assisted living facilities. Without close examination of the type of housing, past research has omitted considerable information about support structures that allow people to live “alone.” While the household roster of older adults living in assisted living facilities would show only one person, their sources of care are far more robust than those of older adults living alone in single-family housing. We included individuals in assisted living in our analysis in other households due to small cell sizes. When possible, future research should acknowledge older adults in assisted living as unique segment of the community-based older adult population. Older adults in assisted living represent an understudied population of older adults who can live with more independence than those in nursing homes but less than those living alone.

Future research on living arrangements for Mexican and Mexican American older adult populations studied here should incorporate additional considerations. First, we used only cross-sectional data, and future research should seek to address causal ordering for living alone in late life. We were unable to capture how the dynamics of living arrangements and cognitive functioning interact for older adults to arrive in late life with dementia living alone. Second, among the older adults who live alone, it is important to consider the role of child proximity. Previous studies suggest that U.S. adults over the age of 65 spend more time living near their children than coresiding (Raymo et al., 2019). Additionally, it is possible that the high rates of living children among those living alone in Mexico do not translate to potential sources of care due to the migration of adult children both within and outside Mexico. Roughly one-quarter of older Mexicans have a migrant child living in the United States and a larger share of children live elsewhere in Mexico (Torres et al., 2018). No research has focused on patterns of migration among children of older Mexican Americans specifically, but it is unlikely that children of Mexican American older adults have similarly high levels of international migration. While our focus is primarily on older adults who live alone, it is important to note that the structure of household extension is different between the United States and Mexico for the oldest old. Previous research indicates that, for Mexican Americans, the majority of household extensions are with adult children (71.2%) or other family (25.2%), with only a small fraction coresiding with nonfamily (3.1%; Prickett & Angel, 2017). Extended households in Mexico tend to be larger and include a greater diversity of family members and grandchildren (Huffman et al., 2019). Most extended households in Mexico for adults over age 85 include grandchildren (55%) and other family (40.8%), while relatively few include nonfamily (2.4%; Huffman et al., 2019). We also note that the inclusion of wealth and homeownership in our regression analysis may be measuring the same underlying concept. This may be especially problematic for the U.S. sample, where eligibility for Medicaid can disincentivize homeownership (Angel et al., 2019). While we analyzed living arrangements for the oldest old with dementia using logistic regression, we did not include the results due to small sample size. Ultimately, we felt that the results from the underpowered regression analysis did not help us understand the demographic profiles of older adults with dementia living alone. Finally, our classification of probable dementia is not a clinical diagnosis of dementia. Clinical diagnoses are required to determine dementia, but we remain confident that our classifications reflect the high levels of cognitive impairment and care need characteristics of dementia.

## Conclusion

Housing and care arrangements for older adults in Mexico differ significantly from those of Mexican Americans. Dementia is associated with higher odds of living alone in the U.S. sample, but not in Mexico. Overall, this may reflect the fact that, while one-person households among older adults in Mexico are increasing, the wide majority of older adults still live in extended households as these arrangements are part of the subsistence strategies families use to support each other and where both generations, older adults and adult children, provide support, such as health and personal care, money, etc., according to gender roles and each generation’s needs and resources (Gomes, 2007). In the future, Mexico may mimic patterns of living arrangements in the United States due to significant population aging trends, decreased mortality, and a lower fertility regime, but the lack of comparable national long-term care policies remains a challenge.

Mexican Americans comprise a diverse array of people from first, to second, to third plus generations living primarily in the American Southwest, the Midwest, and increasingly branching out to other parts of the country (Hernández & Moreno-Fernández, 2018). Mexicans Americans living in the Southwest may be more culturally similar to Mexicans than those living in other regions (Portes & Rumbaut, 2001). The experiences of older Mexican Americans living in the Southwest may offer insight into what Mexican Americans in new migration destinations of the United States will experience in the future.

Despite all efforts to support individual autonomy and the fact that aging in place may be the preferred model for late life, our findings demonstrate that independence may ultimately become impossible for many older individuals with dementia. In the very near future, both the United States and Mexico will face an increasing need for support strategies for older adults with dependency care needs, and in particular for people living with dementia and their family caregivers. While various levels of institutional care could be a solution, both countries should focus on increasing integrated community care strategies to increase the period older adults can live at home. Invariably, increasing life spans and increasing prevalence of chronic degenerative illnesses such as dementia will create growing demands on already strained public budgets and families currently providing the majority of the available support.

## Supplementary Material

igac014_suppl_Supplementary_Material
