# Supplementary figures and images for: Living Arrangements and Dementia Among the Oldest Old: A Comparison of Mexicans and Mexican Americans
Source: Innov Aging. 2022 Mar 17;6(3):igac014. doi: 10.1093/geroni/igac014 (PMC9154059; doi:10.1093/geroni/igac014)

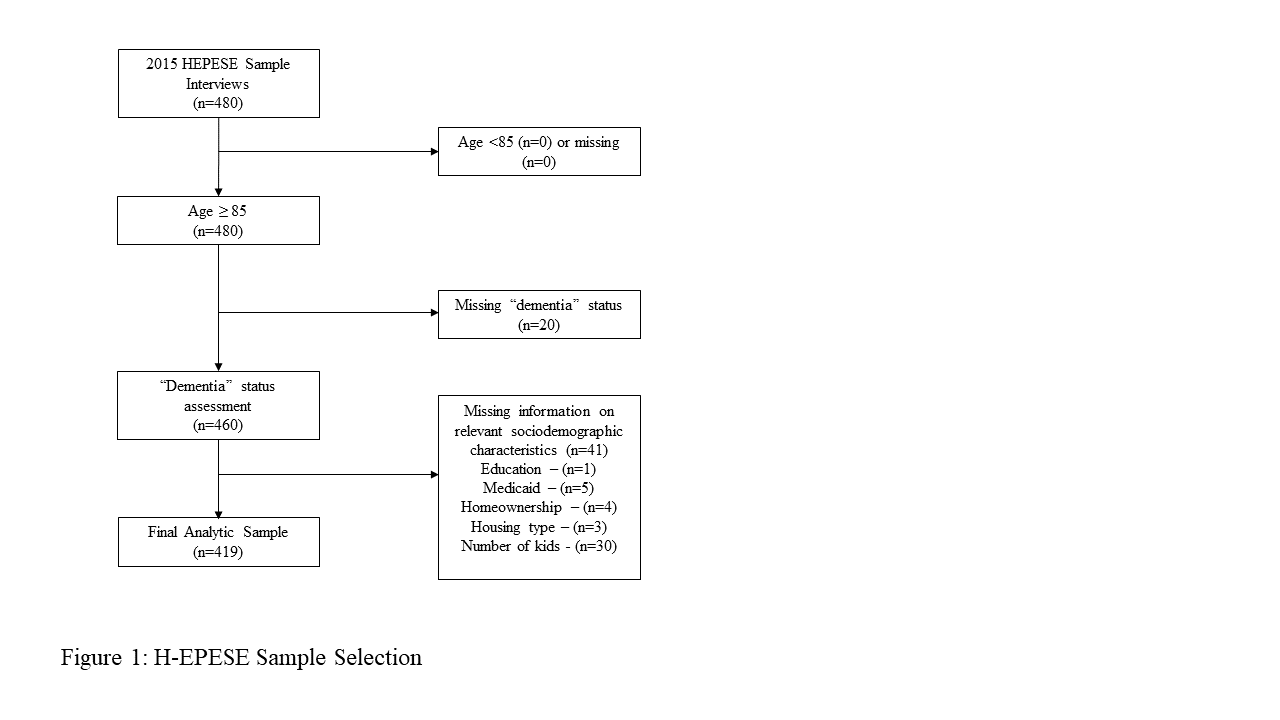

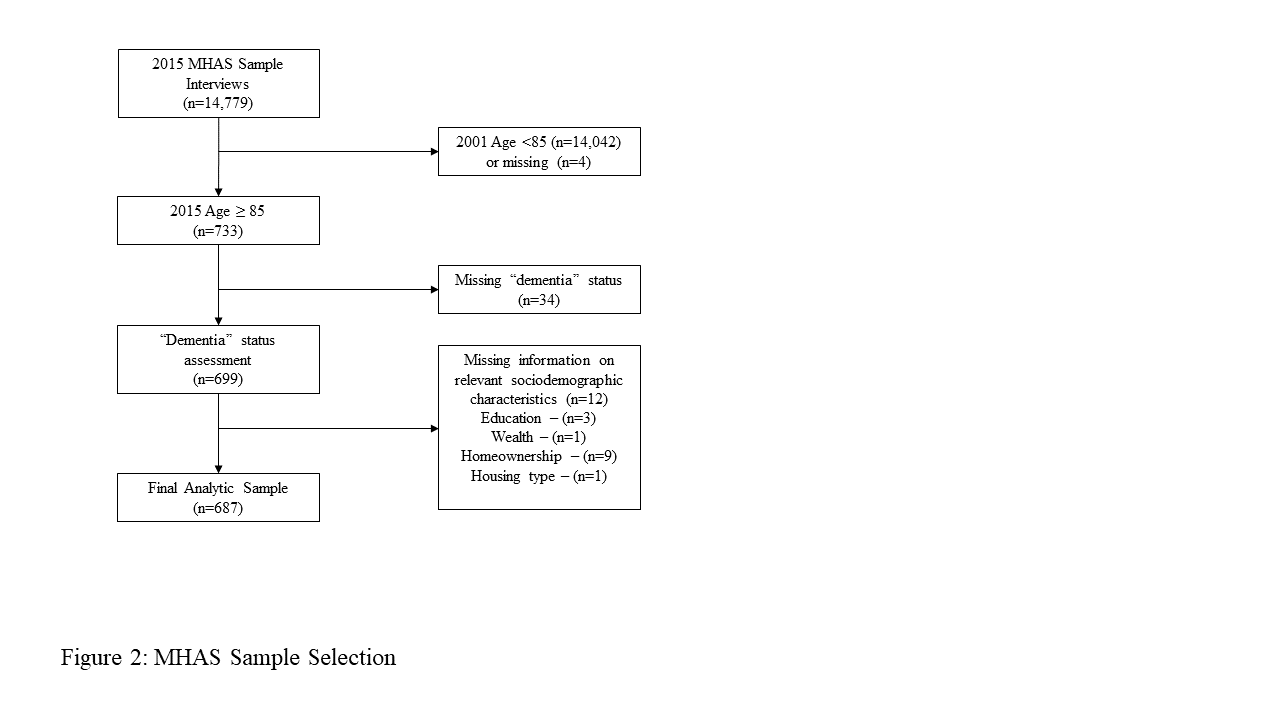

Supplement: igac014_suppl_Supplementary_Material [file igac014_suppl_supplementary_material.docx]
